# Effects of chemical myosin II cross‐bridge inhibitors on eccentric force production in skinned muscle fibres

**DOI:** 10.1113/EP093831

**Published:** 2026-06-01

**Authors:** André Tomalka, Sven Weidner, Christian Rode, Tobias Siebert

**Affiliations:** ^1^ Motion and Exercise Science University of Stuttgart Stuttgart Germany; ^2^ Institute of Sport Science, Department of Biomechanics University of Rostock Rostock Germany; ^3^ Stuttgart Center for Simulation Science University of Stuttgart Stuttgart Germany

**Keywords:** active muscle lengthening, actomyosin inhibition, blebbistatin, contractile behaviour, cross‐bridge block, myosin‐active compounds, small molecule inhibitor

## Abstract

Eccentric muscle contractions are essential for locomotion and daily activities. Force during these contractions depends on both cross‐bridge (XB) and non‐cross‐bridge (non‐XB) elements, including titin, with contributions varying by fibre type and contraction velocity. Despite their importance, mechanisms of force generation during prolonged stretches remain unclear. Chemical XB inhibitors are commonly used to separate XB and non‐XB contributions, but their effects have not been systematically compared under identical conditions. We measured eccentric force in single permeabilised fibres from fast‐twitch rat extensor digitorum longus (EDL) and slow‐twitch soleus (SOL) muscles. Fibres were treated with blebbistatin (Bleb), 2,3‐butanedione monoxime (BDM), or *N*‐benzyl‐*p*‐toluene sulphonamide (BTS) and stretched from 0.85 to 1.3 times optimal length at three velocities (0.01, 0.1 and 1.0 maximal contraction velocity). XB inhibition produced fibre‐type‐dependent effects on early‐ and late‐stretch force (slope_1_, slope_2_) and on end‐of‐stretch force (*s*_e_). Stretch velocity and the type of XB inhibitor influenced *s*_e_ in both SOL and EDL fibres. However, in EDL fibres, *s*_e_ did not differ significantly between XB inhibitors and showed velocity‐dependent effects under Bleb and BTS only, but not under BDM. Stretch‐induced forces were less reduced than isometric forces, indicating substantial stretch‐dependent non‐XB contributions. These results show that eccentric force arises from coordinated XB and non‐XB mechanisms in a fibre‐type‐dependent manner and highlight Bleb as an effective tool for probing these contributions.

## Abstract (highlights)

- **What is the central question of this study?**
  How do cross‐bridge inhibitors – blebbistatin (Bleb), 2,3‐butanedione monoxime (BDM) and *N*‐benzyl‐*p*‐toluene sulphonamide (BTS) – affect isometric and eccentric force in fast‐ and slow‐twitch muscle fibres, and what are the relative contributions of cross‐bridge and non‐cross‐bridge mechanisms?
- **What is the main finding and its importance?**
  Eccentric force is fibre‐type‐dependent and arises from coordinated cross‐bridge (XB) and non‐cross‐bridge (non‐XB) mechanisms, with substantial non‐XB contributions persisting despite XB inhibition. Blebbistatin effectively isolates these contributions in both fibre types, providing a robust tool to probe the mechanisms underlying prolonged eccentric muscle contractions.

## 1 INTRODUCTION

Eccentric muscle contractions, essential for activities like running and jumping, remain poorly understood despite their importance for movement (Hody et al., 2019; Hoppeler, 2016; LaStayo et al., 2014; Tomalka, 2023). Force generation during these contractions involves both cross‐bridge (XB) and non‐cross‐bridge (non‐XB) elements, such as titin (Linke, 2023; Tomalka, 2023), and is modulated by fibre type (Ausoni et al., 1990; Freundt & Linke, 2019; Greaser & Pleitner, 2014; Prado et al., 2005; Ramsey et al., 2010; Weidner et al., 2024), contraction velocity (Charles & Kissane, 2024; Lombardi & Piazzesi, 1990; Pinniger et al., 2006; Weidner et al., 2022) and muscle activation (Araz et al., 2023; Elst et al., 2025). Understanding how these factors interact is essential for explaining the distinctive mechanical behaviour of muscle during eccentric contractions.

**FIGURE A1:**
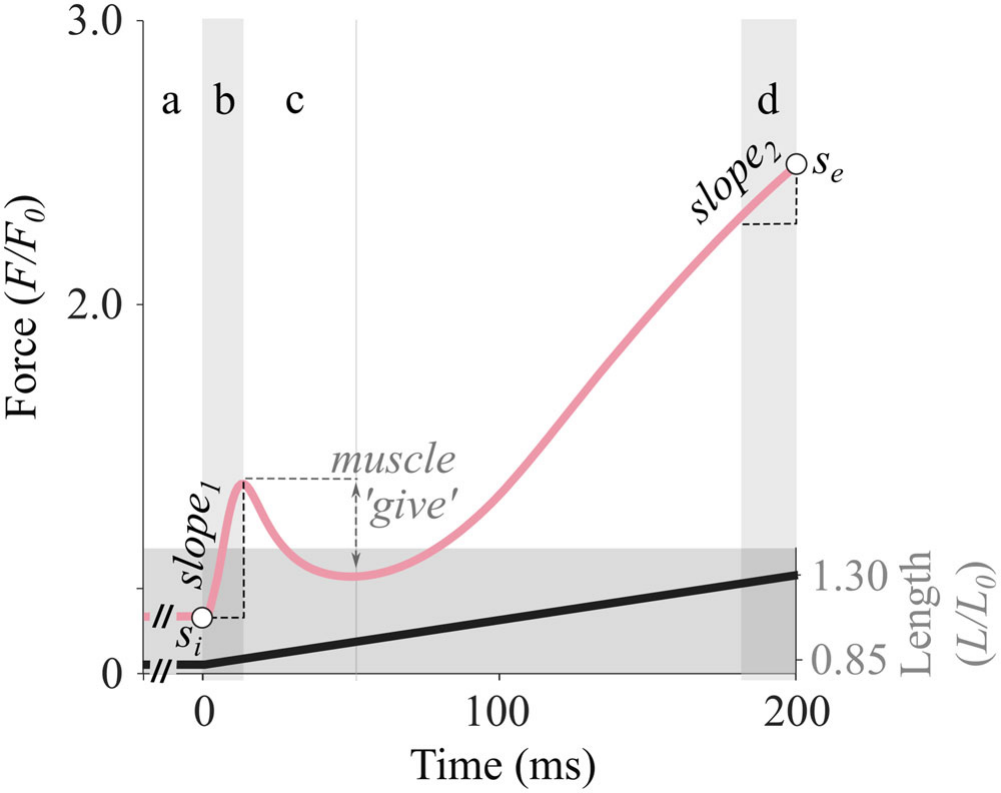
Biphasic force response during stretch. Muscle fibres were isometrically activated (a) before isokinetic ramp stretches from 0.85–1.3*L*_*0*_. Stretch onset produced an early force development (slope_1_; b), followed by a transient drop in force (muscle ‘*give*’; c). Force then redeveloped with an approximately constant, quasi‐linear increase, with slope_2_ (late‐stretch force development, d) determined over the final 5% of the stretch. Open circles indicate forces at stretch onset (*s*_*i*_) and at the end of the stretch (*s*_*e*_). Slopes (Δforce/Δlength) were calculated from linear regression of force–length data. *T* = 0 ms denotes the onset of active lengthening after steady‐state isometric force had been achieved.

During the initial stretch phase, muscle force rises steeply (slope_1_), often reaching a characteristic peak (*s*_2_), with both slope and peak increasing with stretch velocity (Pinniger et al., 2006; Tomalka et al., 2021; Weidner et al., 2022, 2024) (Appendix, Figure A1). This response is typically followed by a transient decrease in force, referred to as muscle ‘*give’*, observed in both whole muscle (Flitney & Hirst, 1978; Till et al., 2008) and fibres (Choi & Widrick, 2010; Griffiths et al., 1980) during rapid active lengthening. During longer stretches, force rises again nearly linearly (Kissane & Askew, 2024; Till et al., 2008; Tomalka et al., 2017; Weidner et al., 2022, 2024), with the late‐phase slope (slope_2_) exceeding predictions from the isometric force–length relationship (Tomalka et al., 2017) and increasing with stretch velocity (Tomalka et al., 2021; Weidner et al., 2022, 2024).

The progressive force enhancement observed during prolonged stretches cannot be fully explained by XB mechanisms described in the classical XB (Huxley, 1957) and sliding filament theories (Huxley & Hanson, 1954; Huxley & Niedergerke, 1954). Beyond optimal muscle length, muscle models predict a decline in XB force as the number of attached XBs decreases. However, recent experimental evidence demonstrates that titin and other non‐XB structures lead to increasing fibre forces during active lengthening, particularly at longer muscle lengths (Leonard & Herzog, 2010; Linke, 2018; Tomalka et al., 2017).

Titin is a giant sarcomeric protein spanning from the Z‐line to the M‐line, interacting with both thin and thick filaments (Granzier & Labeit, 2006; Linke, 2023). It acts as a molecular spring, maintaining sarcomere alignment and resisting overextension during stretch (Hessel et al., 2022; Linke, 2018). Titin stiffness increases with activation and elevated Ca^2^^+^ (Labeit et al., 2003; Powers et al., 2020), contributing to length‐dependent activation (Ait‐Mou et al., 2016) through thick filament mechano‐sensing (Fusi et al., 2016) and modulation of troponin Ca^2^^+^ affinity (Rassier & Månsson, 2025), potentially alongside MyBP‐C (Hessel, Engels et al., 2024, Hessel, Kuehn et al., 2024).

These observations underscore the importance of distinguishing the individual contributions of XB and non‐XB elements to eccentric force generation to refine mechanistic understanding and improve muscle models. However, experimentally separating these contributions remains challenging.

Chemical XB inhibitors provide an experimental approach for probing XB‐ and non‐XB‐mediated force components in skeletal (Higuchi & Takemori, 1989), cardiac (Backx et al., 1994) and smooth muscle (Arner & Malmqvist, 1998). These pharmacological agents target myosin motor proteins and selectively inhibit myosin II ATPase activity, thereby limiting reversible actomyosin XB formation (Cheung et al., 2002; Kovács et al., 2004). Comprehensive overviews of pharmacological modulation of actomyosin interactions are provided by Rassier & Månsson (2025) and Bond et al. (2013).

By suppressing XB mechanics, force generation and shortening velocity are reduced through modulation of actomyosin ATPase activity (Bagni et al., 1992; Fryer et al., 1988; Regnier et al., 1995). XB inhibitors effectively limit the contractile apparatus (Herrmann et al., 1992; Higuchi & Takemori, 1989), allowing detailed investigation of XB‐dependent and non‐XB contributions (Rahman et al., 2018; Rassier & Månsson, 2025). They also provide a basis for potential therapeutic strategies targeting myosin‐related disorders (Jungbluth et al., 2018).

The most commonly used XB inhibitors in muscle physiology are blebbistatin (Bleb), 2,3‐butanedione monoxime (BDM) and *N*‐benzyl‐*p*‐toluene sulphonamide (BTS) (Cheung et al., 2002; Limouze et al., 2004; McKillop et al., 1994). While all these agents suppress active XB force, they act through partially distinct molecular mechanisms, targeting different binding sites (Bond et al., 2013). Consequently, their effects on force generation during eccentric contractions may differ.

Despite their frequent use, systematic investigations of the effects of chemical XB inhibitors on eccentric force remain limited. Most previous studies employed small to moderate stretch amplitudes (1–6% *L*_*0*_) and focused on individual agents such as BDM, BTS and Bleb (Colombini, Nocella, Benelli et al., 2010, 2016; Iwamoto, 2018; Powers et al., 2014; Rassier & Herzog, 2004). These investigations suggest that, under XB inhibition, a substantial fraction of myosin heads remain attached in weakly bound, low‐force states. Biochemical evidence indicates that these inhibitors block the force‐producing transition (from a weakly bound, low‐force state to a strongly bound, high‐force state) (Iwamoto, 2018) rather than actin binding (Herrmann et al., 1992; McKillop et al., 1994; Regnier et al., 1995; Zhao & Kawai, 1994). Consistent with this mechanism, early‐phase eccentric force during small stretches appears to involve an increased population of low‐ or non‐force‐generating XBs (Colombini, Nocella, Bagni et al., 2010; Iwamoto, 2018). The slow decay of XB‐inhibited force during stretch further suggests altered XB attachment–detachment kinetics, highlighting how chemical inhibitors differentially modulate XB dynamics. Moreover, these agents may affect other sarcomeric proteins and intracellular processes, leading to context‐dependent changes in force generation (Iwamoto, 2018; Månsson et al., 2015; Minozzo & Rassier, 2010; Rahman et al., 2018).

Although individual XB inhibitors have been examined in various muscles and protocols (Bagni et al., 1992; Pinniger et al., 2006; Rassier & Herzog, 2004; Shalabi et al., 2017), direct comparisons of multiple inhibitors under identical experimental conditions – particularly during prolonged ramp stretches within the same muscle – are lacking. Such comparisons are critical to determine whether these inhibitors produce similar or distinct functional effects during eccentric lengthening and to clarify how XB inhibition modulates non‐XB force contributions in slow and fast muscles. Slow‐twitch fibres exhibit lower myosin ATPase activity, XB cycling frequency and maximal shortening velocity compared with fast‐twitch fibres (Barclay, 2019; Capitanio et al., 2006; Charles & Kissane, 2024). Differences in non‐XB structures, such as connective tissue and titin isoforms (Greaser & Pleitner, 2014; Prado et al., 2005; Wang et al., 1991; Ward et al., 2020), also contribute to distinct passive properties that influence eccentric force generation (Kissane & Askew, 2024; Siebert et al., 2015). Recently, Weidner et al. (2024) demonstrated fibre type‐specific differences in XB and non‐XB contributions to eccentric force during prolonged stretches.

Despite their extensive use, no study has directly compared the effects of multiple chemical XB inhibitors on eccentric force in fast‐ and slow‐twitch fibres during prolonged stretches across different velocities. Here, we address this gap by investigating eccentric force at the single‐muscle‐fibre level in permeabilised rat extensor digitorum longus (EDL) and soleus (SOL) fibres. Fibres were stretched from 0.85 to 1.3 times their optimal fibre length at velocities of 0.01, 0.1 and 1.0 times maximum contraction velocity (*v*_*0*_). This study aimed to determine: (1) how different chemical XB inhibitors influence eccentric force production and whether these effects depend on fibre type; (2) the extent to which non‐XB structures, such as titin, contribute to force generation under varying mechanical and biochemical conditions; and (3) how fibre type and stretch velocity modulate the interaction between XB‐ and non‐XB‐mediated force components during eccentric contractions.

## 2 METHODS

### 2.1 Ethical approval

All procedures were approved in accordance with German animal protection law (Tierschutzgesetz, §4 (3); Permit Number: T 201_21 ST). This work conforms to the ethical requirements outlined by the journal.

Glycerinated‐skinned single‐fibre segments were isolated from the EDL muscles of five female Wistar rats (age: 8–10 months, weight: 300–350 g) and the SOL of six female Wistar rats (age: 3 months, weight: 428–520 g). The rats were cage‐sedentary, maintained on a 12 h–12 h light–dark cycle, and housed at a temperature of 22°C. A detailed description of muscle fibre handling, preparation, measurement procedures and solutions used is provided in the Appendix.

### 2.2 Experimental protocol

Fibres were activated through calcium diffusion (pCa 4.5) in the presence of ATP, following the protocols described in detail in Tomalka et al. (2017) and Weidner et al. (2024). The experimental protocol included two ‘treatments’ of repeated measurements: the control treatment (without XB inhibition) and the chemical agents treatment (with XB inhibition). In the control treatment, the dynamic force responses of single‐skinned muscle fibres during isokinetic ramp perturbations under physiological conditions were examined. In the chemical agents treatment, the same skinned muscle fibres were subjected to the same ramp perturbations as in the control, but with the addition of specific chemical XB inhibitors to all solutions. In total, three different chemical agents – Bleb (20 µM), BDM (10 mM) and BTS (50 µM for EDL fibres (Pinniger et al., 2005) and 450 µM for SOL fibres (Cheung et al., 2002)) were tested separately in the same experimental protocol. Stock solutions were prepared by dissolving BTS in dimethyl sulfoxide, Bleb in *N,N*‐Dimethylformamide and BDM in distilled water. Saturating concentrations of the chemical inhibitors were selected to ensure maximal active XB inhibition. The purpose of this treatment was to identify potential effects of XB inhibitors on eccentric force responses during prolonged stretch and to assess the non‐XB contributions to muscle force in SOL and EDL muscles. The SOL is a well‐characterised slow‐twitch muscle, with approximately 96% type I fibres, whereas the EDL is predominantly fast‐twitch, with approximately 94.5% type II fibres (18.8% type IIa and 75.7% type IIb) (Soukup et al., 2002). Individual fibre typing was not performed; SOL and EDL fibres were assumed to be slow and fast, respectively.

**FIGURE 1:**
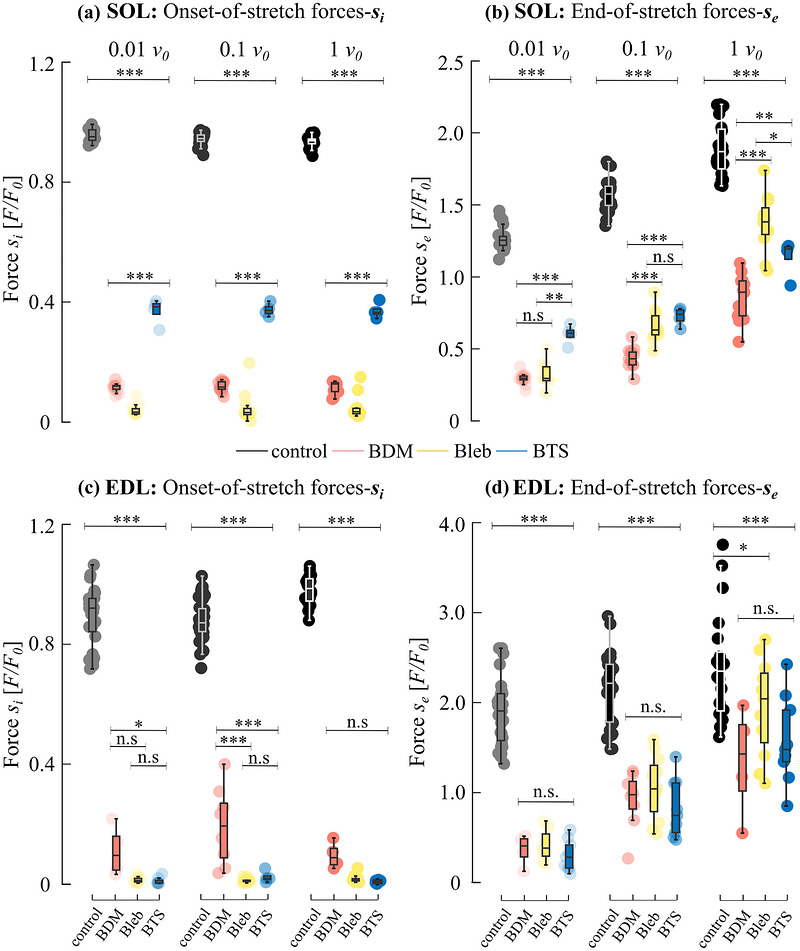
Onset‐of‐stretch and end‐of‐stretch forces in soleus (SOL; a, b) and extensor digitorum longus (EDL; c, d) fibres at stretch velocities of 0.01, 0.1, and 1*v*_*0*_. Forces at stretch onset (*s*_*i*_; a, c) and at the end of stretch (*s*_*e*_; b, d) are shown. Control fibres (grey/black) lacked cross‐bridge (XB) inhibition; treated fibres (red, BDM; yellow, blebbistatin (Bleb); blue, BTS) reveal non‐XB contributions. Boxplots show distributions with individual points overlaid; asterisks denote significant differences: *P < 0.05, **P < 0.01, ***P < 0.001; n.s., not significant. Number of fibres: SOL: 0.01*v*_*0*_ (control: 23, BDM: 13, Bleb: 11, BTS: 4); 0.1*v*_*0*_ (control: 23, BDM: 13, Bleb: 11, BTS: 4); 1*v*_*0*_ (control: 23, BDM: 13, Bleb: 11, BTS: 4). EDL: 0.01*v*_*0*_ (control: 27, BDM: 4, Bleb: 11, BTS: 9); 0.1*v*_*0*_ (control: 35, BDM: 8, Bleb: 11, BTS: 9); 1*v*_*0*_ (control: 20, BDM: 4, Bleb: 11, BTS: 9). These sample sizes apply to all figures (Figures 1, 2, 3, 4, 5, Appendix Figure A2) and tables (Tables 1, 2, 3, 4) throughout the study.

**FIGURE 2:**
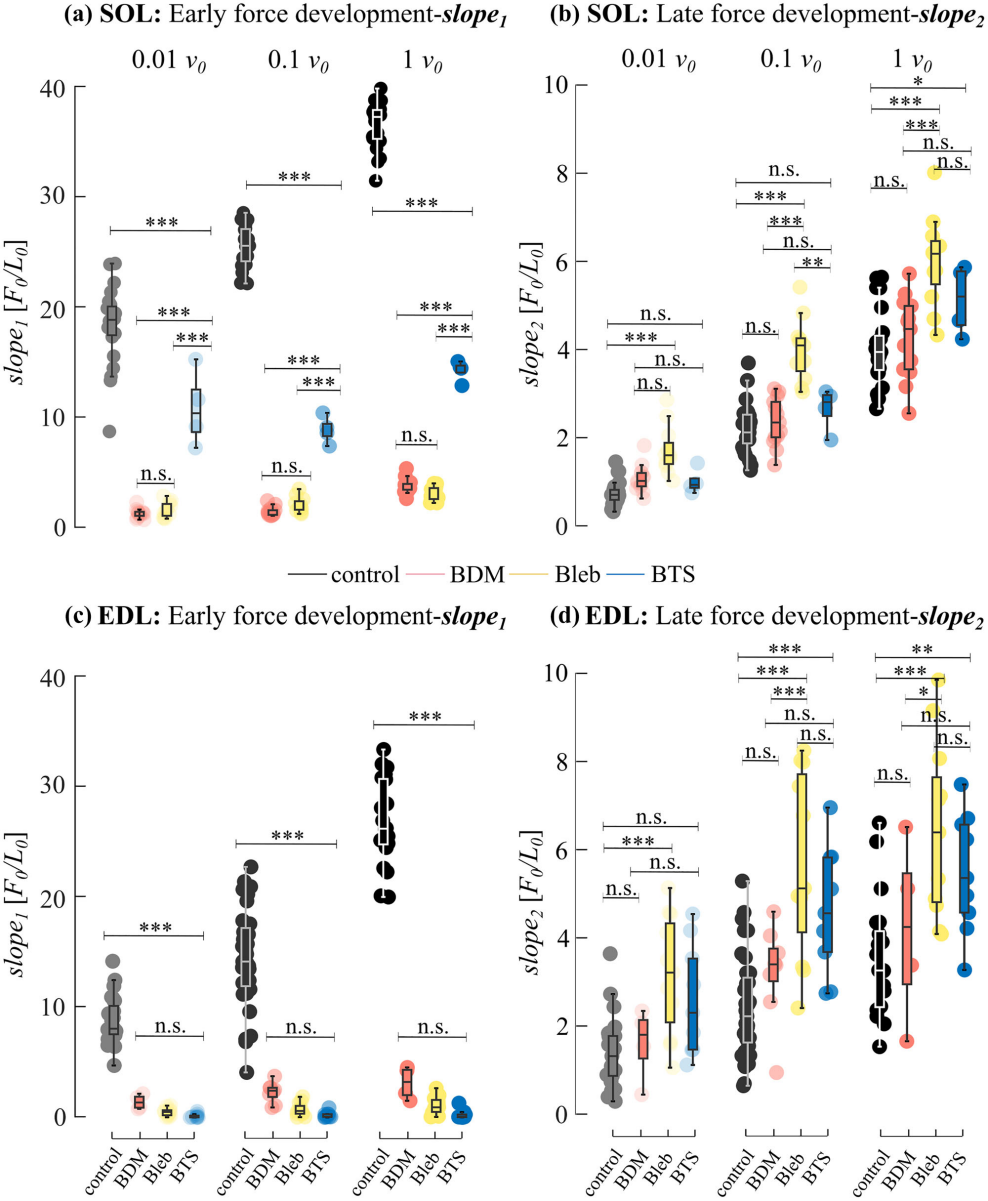
Early and late force development during stretch in soleus (SOL; a, b) and extensor digitorum longus (EDL; c, d) fibres at velocities of 0.01, 0.1, and 1*v*_*0*_. Early‐ (slope_1_; a, c) and late force development over the final 5% of stretch (slope_2_; b, d) are shown. Control fibres (grey/black) lacked XB inhibition; treated fibres (red, BDM; yellow, Bleb; blue, BTS) reveal non‐XB contributions. Boxplots show distributions with individual points; asterisks denote significant differences: *P < 0.05, **P < 0.01, ***P < 0.001; n.s., not significant. Fibre numbers per velocity and treatment are identical to those reported in Figures 1, 2, 3, 4, 5.

**FIGURE 3:**
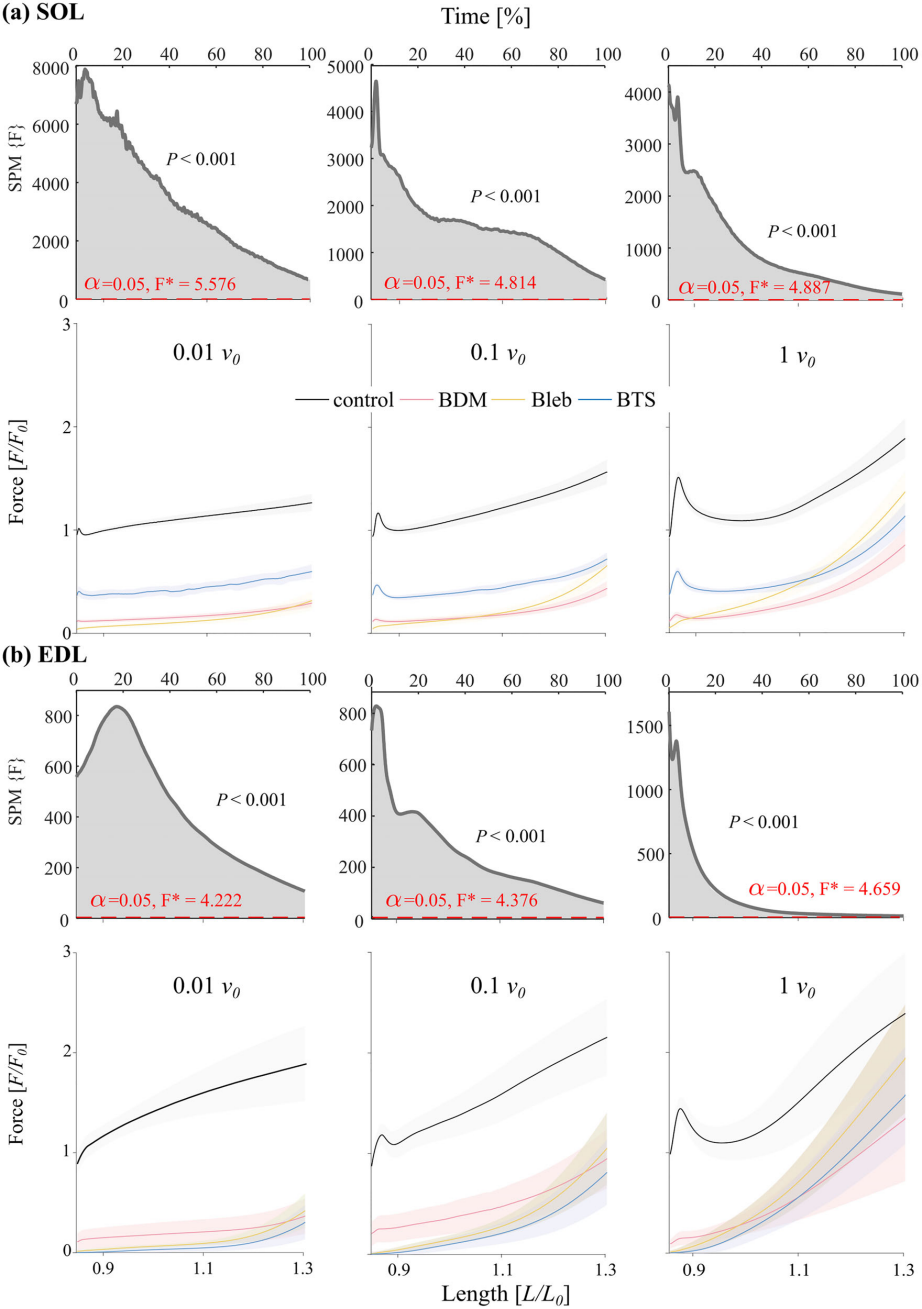
Eccentric force development of single muscle fibres in the soleus (SOL, a; top) and extensor digitorum longus (EDL, b; bottom) under control and cross‐bridge (XB) inhibition conditions. Force responses were measured during long (45% *L*_*0*_, from 0.85 to 1.3 *L*_*0*_) isokinetic stretches at 0.01, 0.1 and 1*v*_*0*_. Control trials are shown in black; XB inhibitors are BDM (red), Bleb (yellow), and BTS (blue). Solid lines indicate mean normalized force (*F/F*_*0*_), and shaded areas represent standard deviation. Top panels: SPM{*F*} analyses showing regions of significant differences among the three XB inhibitors over normalized fibre length (*L*/*L*_*0*_) and time (% of stretch). Grey‐shaded areas indicate statistically significant regions, and the red dashed line denotes the critical *F*‐value at α = 0.05. Bottom panels: force–length curves for each inhibitor at each velocity. Fibre numbers per velocity and treatment are identical to those reported in Figures 1, 2, 3, 4, 5.

**FIGURE 4:**
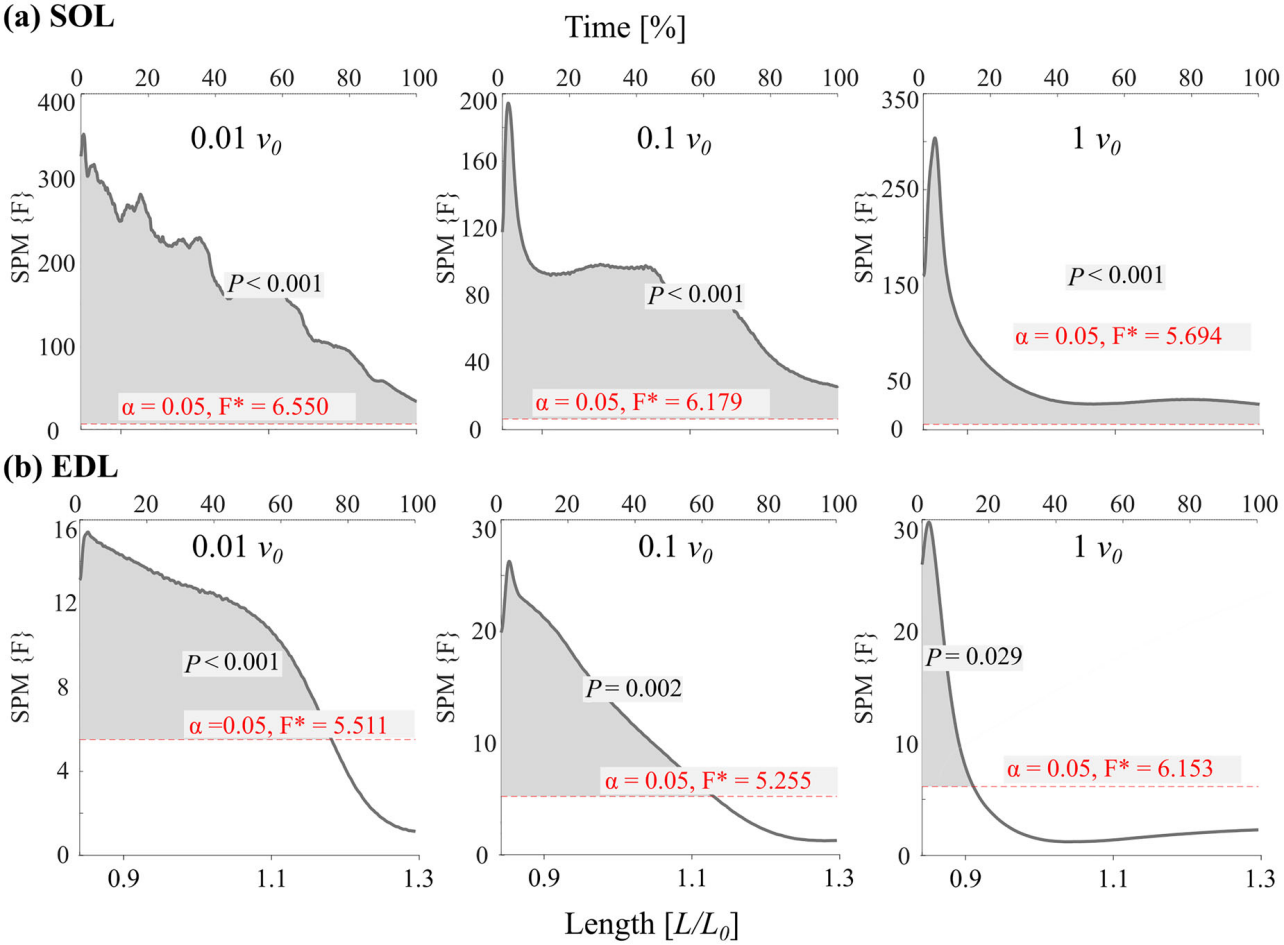
SPM{*F*} analyses of cross‐bridge (XB) inhibition effects on force responses in single muscle fibres from the soleus (SOL, a) and extensor digitorum longus (EDL, b). Shown are results for stretches at 0.01*v*_*0*_ (left), 0.1*v*_*0*_ (middle) and 1*v*_*0*_ (right) under BDM, Bleb and BTS. Grey‐shaded areas represent SPM{*F*} statistics over normalized fibre length (*L*/*L*_*0*_) and time (% of stretch). The red dashed line indicates the critical *F*‐value (α = 0.05). Reported *P*‐values correspond to overall ANOVA significance across inhibitors. Fibre numbers per velocity and treatment are identical to those reported in Figures 1, 2, 3, 4, 5.

**FIGURE 5:**
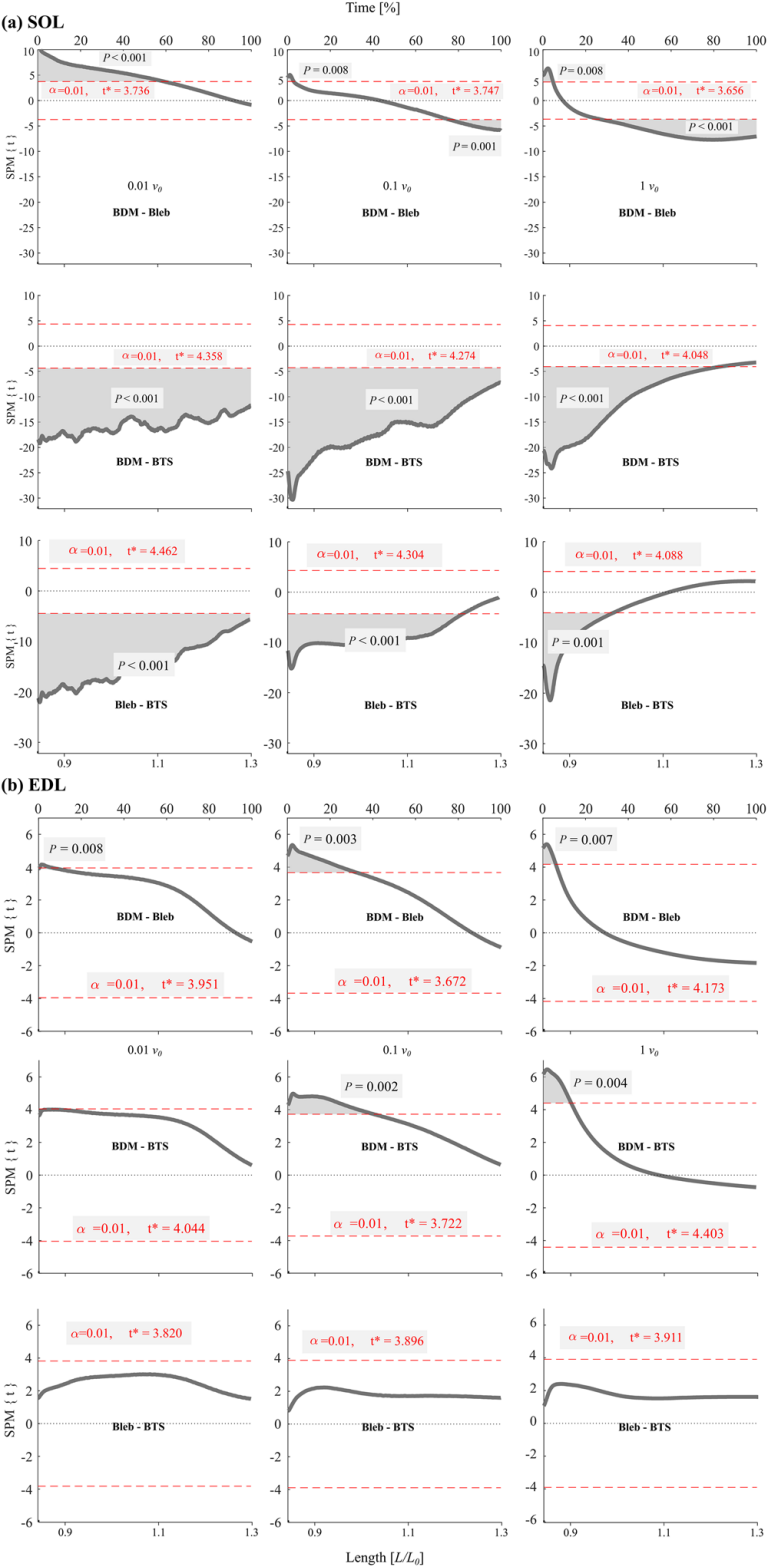
SPM{*t*} *post hoc* pairwise comparisons of force responses across cross‐bridge (XB) inhibitors in single muscle fibres from the soleus (SOL, a) and extensor digitorum longus (EDL, b). Each panel presents comparisons among BDM–Bleb, BDM–BTS and Bleb–BTS at three stretch velocities: 0.01*v*_*0*_ (left), 0.1*v*_*0*_ (middle) and 1*v*_*0*_ (right). Dark grey curves depict the *t*‐statistic as a function of normalized length (*L/L*_*0*_) and time (0–100% of the stretch phase). The horizontal dashed red line represents the critical threshold (t*) for significance at α = 0.01 (Bonferroni‐corrected), and grey‐shaded regions indicate intervals where significant differences between inhibitors were detected. Fibre numbers per velocity and treatment are identical to those reported in Figures 1, 2, 3, 4, 5.

**FIGURE A2:**
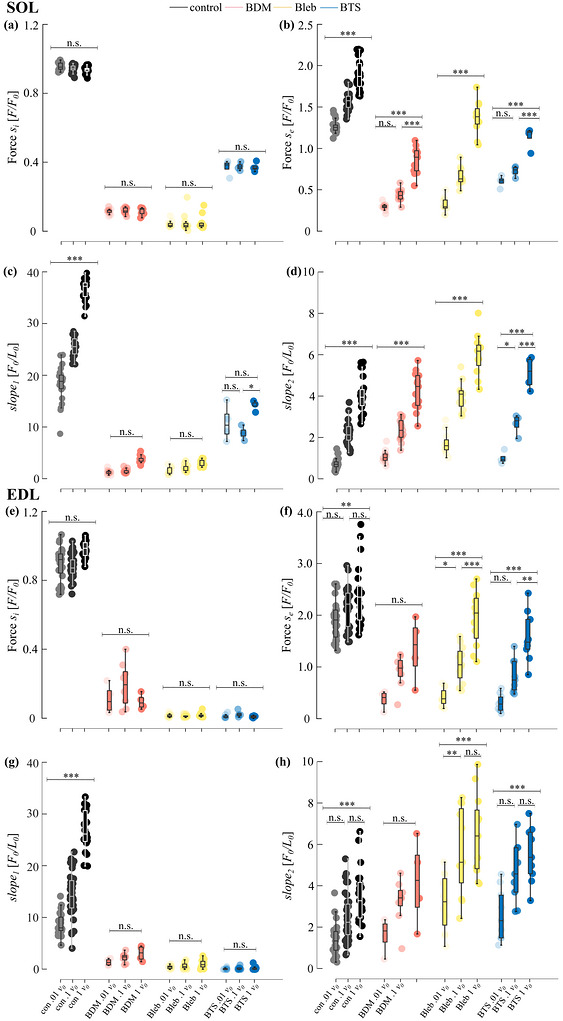
Velocity‐dependent effects of cross‐bridge (XB) inhibition on stretch‐induced force characteristics in soleus (SOL; a–d) and extensor digitorum longus (EDL; e–h) single fibres at 0.01, 0.1 and 1 *v*_*0*_. Panels show onset‐of‐stretch forces (*s*_*i*_) and end‐of‐stretch forces (*s*_*e*_), and early‐ (slope_1_) and late‐stretch (slope_2_; final 5% of stretch) force development. Control fibres (grey/black) were untreated; XB‐inhibited fibres were treated with BDM (red), Bleb (yellow), or BTS (blue) to reveal non‐XB contributions. Boxplots depict distributions with individual data points overlaid. Asterisks indicate significant differences (*P < 0.05, **P < 0.01, ***P < 0.001; n.s., not significant). Number of fibres: SOL: 0.01*v*_*0*_ (control: 23, BDM: 13, Bleb: 11, BTS: 4); 0.1*v*_*0*_ (control: 23, BDM: 13, Bleb: 11, BTS: 4); 1*v*_*0*_ (control: 23, BDM: 13, Bleb: 11, BTS: 4) EDL: 0.01*v*_*0*_ (control: 27, BDM: 4, Bleb: 11, BTS: 9); 0.1*v*_*0*_ (control: 35, BDM: 8, Bleb: 11, BTS: 9); 1*v*_*0*_ (control: 20, BDM: 4, Bleb: 11, BTS: 9).

Activated single‐skinned fibres were stretched from 0.85 *L*_0_ to 1.3*L*_*0*_. The perturbations were conducted at different stretch velocities of 0.01, 0.1 and 1*v*_*0*_. The maximum shortening velocities of skinned EDL (2.42 ± 0.08*L*_*0*_ s^−1^; *n* = 5 fibres) and SOL (0.47 ± 0.11*L*_*0*_ s^−1^; *n* = 6 fibres) muscle fibres from adult male Wistar rats were determined in separate sets of muscle fibres, as described in Weidner et al. (2022) and Weidner et al. (2024), respectively. The results were consistent with previously reported literature values (Degens et al., 1998; Tomalka et al., 2017). Experiments were first performed under control conditions and subsequently under the XB‐inhibited condition. Within each treatment condition, the order of the ramp protocol was randomised to minimise potential bias and systematic effects. Sample sizes were as follows: SOL: 0.01, 0.1, 1*v*_*0*_ (control: 23, BDM: 13, Bleb: 11, BTS: 4 for all velocities); EDL: 0.01*v*_*0*_ (control: 27, BDM: 4, Bleb: 11, BTS: 9), 0.1*v*_*0*_ (control: 35, BDM: 8, Bleb: 11, BTS: 9), 1*v*_*0*_ (control: 20, BDM: 4, Bleb: 11, BTS: 9). These sample sizes apply to all figures (Figures 1, 2, 3, 4, 5, Appendix Figure A2) and tables (Tables 1, 2, 3, 4).

**TABLE 1:** Velocity‐dependent effects of cross‐bridge inhibition on muscle force parameters in SOL.

Parameter	Velocity	Treatment	Mean ± SD (*n*)	Comparison	Mean diff	*t*	Cohen's *d*	*P*_bonf_
***s*_i_ (*F/F*_0_**)	0.01*v*_0_	Control	0.96 ± 0.02 (23)	BDM vs. Control	−0.838	−92.63	−32.14	<0.001***
		Bleb	0.04 ± 0.02 (11)	Bleb vs. Control	−0.915	−95.73	−35.09	<0.001***
		BTS	0.37 ± 0.04 (4)	BTS vs. Control	−0.586	−41.45	−22.45	<0.001***
		BDM	0.12 ± 0.02 (13)	BDM vs. BTS	−0.253	−16.94	−9.69	<0.001***
				Bleb vs. BTS	0.33	21.65	12.64	<0.001***
				Bleb vs. BDM	0.08	7.21	2.95	<0.001***
	0.1*v*_0_	Control	0.94 ± 0.02 (23)	BDM vs. Control	−0.82	−91.06	−31.60	<0.001***
		Bleb	0.05 ± 0.05 (11)	Bleb vs. Control	−0.89	−93.54	−34.29	<0.001***
		BTS	0.38 ± 0.02 (4)	BTS vs. Control	−0.57	−40.26	−21.81	<0.001***
		BDM	0.12 ± 0.02 (13)	BDM vs. BTS	−0.26	−17.12	−9.79	<0.001***
				Bleb vs. BTS	0.33	21.37	12.48	<0.001***
				Bleb vs. BDM	0.07	6.57	2.69	<0.001***
	1*v*_0_	Control	0.93 ± 0.02 (23)	BDM vs. Control	−0.82	−90.58	−31.43	<0.001***
		Bleb	0.05 ± 0.04 (11)	Bleb vs. Control	−0.88	−92.47	−33.90	<0.001***
		BTS	0.37 ± 0.03 (4)	BTS vs. Control	−0.56	−39.85	−21.59	<0.001***
		BDM	0.12 ± 0.02 (13)	BDM vs. BTS	−0.26	−17.21	−9.84	<0.001***
				Bleb vs. BTS	0.321	21.08	12.31	<0.001***
				Bleb vs. BDM	0.064	6.03	2.47	<0.001***
***s*_e_ (*F/F*_0_)**	0.01*v*_0_	Control	1.27 ± 0.08 (23)	BDM vs. Control	−0.97	21.97	7.62	<0.001***
		Bleb	0.32 ± 0.09 (11)	Bleb vs. Control	−0.95	20.30	7.44	<0.001***
		BTS	0.60 ± 0.07 (4)	BTS vs. Control	−0.67	9.64	5.22	<0.001***
		BDM	0.29 ± 0.04 (13)	BDM vs. Bleb	−0.02	−0.45	−0.18	1.000
				BDM vs. BTS	−0.31	−4.20	−2.40	<0.001***
				Bleb vs. BTS	−0.28	−3.80	−2.22	0.001**
	0.1*v*_0_	Control	1.57 ± 0.12 (23)	BDM vs. Control	−1.13	25.57	8.87	<0.001***
		Bleb	0.66 ± 0.11 (11)	Bleb vs. Control	−0.91	19.43	7.12	<0.001***
		BTS	0.72 ± 0.06 (4)	BTS vs. Control	−0.85	12.23	6.63	<0.001***
		BDM	0.44 ± 0.07 (13)	BDM vs. Bleb	−0.22	−4.27	−1.75	<0.001***
				BDM vs. BTS	−0.29	−3.93	−2.25	<0.001***
				Bleb vs. BTS	−0.06	−0.85	−0.50	1.000
	1*v*_0_	Control	1.89 ± 0.19 (23)	BDM vs. Control	−1.03	23.37	8.11	<0.001***
		Bleb	1.37 ± 0.20 (11)	Bleb vs. Control	−0.52	11.08	4.06	<0.001***
		BTS	1.14 ± 0.13 (4)	BTS vs. Control	−0.75	10.89	5.90	<0.001***
		BDM	0.85 ± 0.16 (13)	BDM vs. Bleb	−0.52	−9.88	−4.05	<0.001***
				BDM vs. BTS	−0.28	−3.87	−2.21	0.001**
				Bleb vs. BTS	0.23	3.15	1.84	0.012*

*Note*: Means ± SD, *n* represents the number of values for isometric onset‐of‐stretch forces (*s*_*i*_), and end‐of‐stretch (*s*_*e*_) across control and XB inhibitor treatments (BDM, Bleb, BTS) at 0.01, 0.1, and 1*v*_*0*_. For calculation of these characteristic parameters, refer to ‘Data processing’ (Methods). *Post hoc* pairwise comparisons were performed within each velocity condition. Statistical results include mean differences, *t*‐values, Cohen's *d* and Bonferroni‐adjusted *P*‐values. (*P < 0.05, **P < 0.01, ***P < 0.001).

**TABLE 2:** Velocity‐dependent effects of cross‐bridge inhibition on slopes in SOL.

Parameter	Velocity	Treatment	Mean ± SD (*n*)	Comparison	Mean diff	*t*	Cohen's *d*	*P*_bonf_
**slope_1_ (*F*_0_ */L*_0_)**	0.01*v*_0_	Control	18.33 ± 3.47 (23)	BDM vs. Control	17.07	24.68	8.57	<0.001***
		Bleb	1.6 ± 0.7 (11)	Bleb vs. Control	16.73	22.90	8.40	<0.001***
		BTS	10.78 ± 3.47 (4)	BTS vs. Control	7.55	6.99	3.79	<0.001***
		BDM	1.26 ± 0.41 (13)	BDM vs. Bleb	−0.34	−0.42	−0.17	1.000
				BDM vs. BTS	−9.53	−8.36	−4.78	<0.001***
				Bleb vs. BTS	−9.19	−7.89	−4.61	<0.001***
	0.1*v*_0_	Control	25.38 ± 1.96 (23)	BDM vs. Control	23.95	34.64	12.02	<0.001***
		Bleb	2.05 ± 0.69 (11)	Bleb vs. Control	23.34	31.94	11.71	<0.001***
		BTS	8.84 ± 1.24 (4)	BTS vs. Control	16.55	15.33	8.30	<0.001***
		BDM	1.43 ± 0.41 (13)	BDM vs. Bleb	−0.62	−0.76	−0.31	1.000
				BDM vs. BTS	−7.40	−6.50	−3.72	<0.001***
				Bleb vs. BTS	−6.79	−5.83	−3.41	<0.001***
	0.1*v*_0_	Control	37.07 ± 2.56 (23)	BDM vs. Control	33.31	48.17	16.72	<0.001***
		Bleb	3.07 ± 0.65 (11)	Bleb vs. Control	34.00	46.53	17.06	<0.001***
		BTS	14.2 ± 0.95 (4)	BTS vs. Control	22.87	21.18	11.47	<0.001***
		BDM	3.75 ± 0.68 (13)	BDM vs. Bleb	0.68	0.84	0.34	1.000
				BDM vs. BTS	−10.45	−9.17	−5.24	<0.001***
				Bleb vs. BTS	−11.13	−9.56	−5.58	<0.001***
**slope_2_ (*F*_0_ */L*_0_)**	0.01*v*_0_	Control	0.75 ± 0.27 (23)	BDM vs. Control	−0.32	−1.40	−0.49	0.977
		Bleb	1.72 ± 0.55 (11)	Bleb vs. Control	−0.97	−4.00	−1.47	<0.001***
		BTS	1.01 ± 0.29 (4)	BTS vs. Control	−0.26	−0.73	−0.39	1.000
		BDM	1.07 ± 0.3 (13)	BDM vs. Bleb	−0.65	−2.39	−0.98	0.109
				BDM vs. BTS	0.06	0.16	0.09	1.000
				Bleb vs. BTS	0.71	1.84	1.07	0.409
	0.1 *v*_0_	Control	2.21 ± 0.58 (23)	BDM vs. Control	−0.16	−0.68	−0.24	1.000
		Bleb	4.0 ± 0.72 (11)	Bleb vs. Control	−1.79	−7.39	−2.71	<0.001***
		BTS	2.65 ± 0.49 (4)	BTS vs. Control	−0.45	−1.24	−0.67	1.000
		BDM	2.36 ± 0.54 (13)	BDM vs. Bleb	−1.64	−6.04	−2.47	<0.001***
				BDM vs. BTS	−0.29	−0.77	−0.44	1.000
				Bleb vs. BTS	1.35	3.48	2.03	0.004**
	1 *v*_0_	Control	4.06 ± 0.85 (23)	BDM vs. Control	−0.21	−0.90	−0.31	1.000
		Bleb	6.0 ± 1.03 (11)	Bleb vs. Control	−1.94	−8.00	−2.93	<0.001***
		BTS	5.13 ± 0.8 (4)	BTS vs. Control	−1.07	−2.98	−1.62	0.020*
		BDM	4.26 ± 0.92 (13)	BDM vs. Bleb	−1.73	−6.40	−2.62	<0.001***
				BDM vs. BTS	−0.86	−2.28	−1.30	0.145
				Bleb vs. BTS	0.87	2.26	1.32	0.153

Means ± SD, *n* represents the number of values for initial stretch (slope_1_), and late stretch (slope_2_) across control and XB inhibitor treatments (BDM, Bleb, BTS) at 0.01, 0.1 and 1*v*_*0*_. For calculation of these characteristic parameters, refer to ‘Data processing’ (Methods). *Post hoc* pairwise comparisons were performed within each velocity condition. Statistical results include mean differences, *t*‐values, Cohen's *d* and Bonferroni‐adjusted *P*‐values (*P < 0.05, **P < 0.01, ***P < 0.001).

**TABLE 3:** Velocity‐dependent effects of cross‐bridge inhibition on muscle force parameters in EDL.

Parameter	Velocity	Treatment	Mean ± SD (*n*)	Comparison	Mean diff	*t*	Cohen's *d*	*P*_bonf_
***s*_i_ (*F/F*_0_)**	0.01*v*_0_	Control	0.89 ± 0.10 (27)	BDM vs. Control	−0.78	−22.06	−11.82	<0.001***
		Bleb	0.01 ± 0.01 (11)	Bleb vs. Control	−0.88	−37.11	−13.27	<0.001***
		BTS	0.00 ± 0.03 (9)	BTS vs. Control	−0.89	−35.01	−13.48	<0.001***
		BDM	0.11 ± 0.09 (4)	BDM vs. BTS	0.11	2.76	1.66	0.039*
				BDM vs. Bleb	0.10	2.49	1.45	0.083
				Bleb vs. BTS	−0.01	−0.45	−0.20	1.000
	0.1*v*_0_	Control	0.88 ± 0.07 (35)	BDM vs. Control	−0.69	−26.51	−10.39	<0.001***
		Bleb	0.01 ± 0.01 (11)	Bleb vs. Control	−0.87	−37.95	−13.12	<0.001***
		BTS	0.01 ± 0.02 (9)	BTS vs. Control	−0.87	−35.31	−13.20	<0.001***
		BDM	0.19 ± 0.13 (8)	BDM vs. BTS	0.19	5.78	2.81	<0.001***
				BDM vs. Bleb	0.18	5.88	2.73	<0.001***
				Bleb vs. BTS	−0.01	−0.18	−0.08	1.000
	1*v*_0_	Control	0.99 ± 0.06 (20)	BDM vs. Control	−0.89	−24.61	−13.48	<0.001***
		Bleb	0.01 ± 0.02 (11)	Bleb vs. Control	−0.97	−39.20	−14.72	<0.001***
		BTS	0.01 ± 0.01 (9)	BTS vs. Control	−0.98	−36.92	−14.82	<0.001***
		BDM	0.1 ± 0.05 (4)	BDM vs. BTS	0.09	2.23	1.34	0.163
				BDM vs. Bleb	0.08	2.12	1.24	0.215
				Bleb vs. BTS	−0.01	−0.23	−0.10	1.000
***s*_e_ (*F/F*_0_)**	0.01*v*_0_	Control	1.89 ± 0.37 (27)	BDM vs. Control	−1.53	−6.93	−3.71	<0.001***
		Bleb	0.42 ± 0.17 (11)	Bleb vs. Control	−1.47	−10.02	−3.59	<0.001***
		BTS	0.30 ± 0.17 (9)	BTS vs. Control	−1.59	−10.04	−3.87	<0.001***
		BDM	0.37 ± 0.18 (4)	BDM vs. Bleb	−0.05	−0.22	−0.13	1.000
				BDM vs. BTS	0.06	0.25	0.15	1.000
				Bleb vs. BTS	0.12	0.62	0.28	1.000
	0.1*v*_0_	Control	2.16 ± 0.38 (35)	BDM vs. Control	−1.24	−7.71	−3.02	<0.001***
		Bleb	1.06 ± 0.36 (11)	Bleb vs. Control	−1.10	−7.74	−2.68	<0.001***
		BTS	0.82 ± 0.32 (9)	BTS vs. Control	−1.34	−8.72	−3.26	<0.001***
		BDM	0.92 ± 0.32 (8)	BDM vs. Bleb	−0.14	−0.75	−0.35	1.000
				BDM vs. BTS	0.10	0.49	0.24	1.000
				Bleb vs. BTS	0.24	1.30	0.58	1.000
	1*v*_0_	Control	2.39 ± 0.60 (20)	BDM vs. Control	−1.04	−4.64	−2.54	<0.001***
		Bleb	1.95 ± 0.54 (11)	Bleb vs. Control	−0.44	−2.87	−1.08	0.028*
		BTS	1.58 ± 0.48 (9)	BTS vs. Control	−0.81	−4.91	−1.97	<0.001***
		BDM	1.35 ± 0.62 (4)	BDM vs. Bleb	−0.60	−2.51	−1.46	0.080
				BDM vs. BTS	−0.23	−0.95	−0.57	1.000
				Bleb vs. BTS	0.37	1.99	0.89	0.293

Means ± SD, *n* represents the number of values for isometric onset‐of‐stretch forces (*s*_*i*_), and end‐of‐stretch forces (*s*_*e*_) across control and XB inhibitor treatments (BDM, Bleb, BTS) at 0.01, 0.1 and 1*v*_*0*_. For calculation of these characteristic parameters, refer to ‘Data processing’ (Methods). *Post hoc* pairwise comparisons were performed within each velocity condition. Statistical results include mean differences, *t*‐values, Cohen's *d* and Bonferroni‐adjusted *P*‐values (*P < 0.05, **P < 0.01, ***P < 0.001).

**TABLE 4:** Velocity‐dependent effects of cross‐bridge inhibition on slopes in EDL.

Parameter	Velocity	Treatment	Mean ± SD (*n*)	Comparison	Mean diff	*t*	Cohen's *d*	*P*_bonf_
**slope_1_ (*F*_0_ */L*_0_)**	0.01*v*_0_	Control	8.84 ± 2.07 (27)	BDM vs. Control	7.41	4.99	2.67	<0.001***
		Bleb	0.49 ± 0.36 (11)	Bleb vs. Control	8.34	8.42	3.01	<0.001***
		BTS	0.13 ± 0.19 (9)	BTS vs. Control	8.71	8.17	3.14	<0.001***
		BDM	1.43 ± 0.67 (4)	BDM vs. Bleb	0.93	0.58	0.34	1.000
				BDM vs. BTS	1.30	0.78	0.47	1.000
				Bleb vs. BTS	0.37	0.29	0.13	1.000
	0.1*v*_0_	Control	14.61 ± 4.49 (35)	BDM vs. Control	12.34	11.37	4.45	<0.001***
		Bleb	0.73 ± 0.61 (11)	Bleb vs. Control	13.88	14.49	5.01	<0.001***
		BTS	0.23 ± 0.28 (9)	BTS vs. Control	14.38	13.89	5.19	<0.001***
		BDM	2.27 ± 0.92 (8)	BDM vs. Bleb	1.54	1.20	0.56	1.000
				BDM vs. BTS	2.04	1.52	0.74	0.791
				Bleb vs. BTS	0.50	0.40	0.18	1.000
	0.1*v*_0_	Control	26.81 ± 3.95 (20)	BDM vs. Control	23.67	15.60	8.54	<0.001***
		Bleb	1.1 ± 0.82 (11)	Bleb vs. Control	25.71	24.71	9.28	<0.001***
		BTS	0.27 ± 0.42 (9)	BTS vs. Control	26.54	23.86	9.58	<0.001***
		BDM	3.14 ± 1.5 (4)	BDM vs. Bleb	2.03	1.26	0.73	1.000
				BDM vs. BTS	2.87	1.72	1.04	0.523
				Bleb vs. BTS	0.84	0.67	0.30	1.000
**slope_2_ (*F*_0_ */L*_0_)**	0.01*v*_0_	Control	1.31 ± 0.85 (27)	BDM vs. Control	−0.31	−0.43	−0.23	1.000
		Bleb	3.18 ± 1.36 (11)	Bleb vs. Control	−1.87	−3.85	−1.38	0.001**
		BTS	2.62 ± 1.26 (9)	BTS vs. Control	−1.31	−2.51	−0.97	0.079
		BDM	1.62 ± 0.84 (4)	BDM vs. Bleb	−1.56	−1.96	−1.15	0.309
				BDM vs. BTS	−1.00	−1.22	−0.73	1.000
				Bleb vs. BTS	0.56	0.92	0.41	1.000
	0.1*v*_0_	Control	2.47 ± 1.16 (35)	BDM vs. Control	−0.78	−1.46	−0.57	0.887
		Bleb	5.7 ± 2.13 (11)	Bleb vs. Control	−3.24	−6.88	−2.38	<0.001***
		BTS	4.65 ± 1.44 (9)	BTS vs. Control	−2.18	−4.29	−1.60	<0.001***
		BDM	3.24 ± 1.1 (8)	BDM vs. Bleb	−2.46	−3.89	−1.81	<0.001***
				BDM vs. BTS	−1.41	−2.13	−1.03	0.212
				Bleb vs. BTS	1.05	1.72	0.78	0.521
	1*v*_0_	Control	3.55 ± 1.33 (20)	BDM vs. Control	−0.64	−0.86	−0.47	1.000
		Bleb	6.48 ± 2.0 (11)	Bleb vs. Control	−2.93	−5.74	−2.15	<0.001***
		BTS	5.51 ± 1.36 (9)	BTS vs. Control	−1.96	−3.59	−1.44	0.003**
		BDM	4.19 ± 2.11 (4)	BDM vs. Bleb	−2.29	−2.88	−1.68	0.027*
				BDM vs. BTS	−1.32	−1.61	−0.97	0.652
				Bleb vs. BTS	0.97	1.59	0.71	0.687

Means ± SD, *n* represents the number of values for initial stretch (slope_1_), and late stretch (slope_2_) across control and XB inhibitor treatments (BDM, Bleb, BTS) at 0.01, 0.1 and 1*v*_*0*_. For the calculation of these characteristic parameters, refer to ‘Data processing’ (Methods). *Post hoc* pairwise comparisons were performed within each velocity condition. Statistical results include mean differences, *t*‐values, Cohen's *d* and Bonferroni‐adjusted *P*‐values (*P < 0.05, **P < 0.01, ***P < 0.001).

### 2.3 Data processing

Force and length signals were sampled at 1 kHz using real‐time software (600A, Aurora Scientific) and an A/D Interface (604A, Aurora Scientific). Data were analysed using a custom‐written MATLAB script (version R2024b, The MathWorks, Natick, MA, USA). Unless stated otherwise, forces were normalised to the individual maximal force (*F/F*_*0*_), shortening velocity to the fibre‐specific maximal contraction velocity (*v/v*_*0*_), and the fibre length to the optimal fibre length (*L*/*L*_*0*_). Two force values (*s*_*i*_ and *s*_*e*_) and two force slopes (slope_1_ and slope_2_) were quantified following Weidner et al. (2022, 2024) (Appendix, Figure A1). Specifically, *s*_*i*_ was the force at stretch onset (isometric force), *s*_*e*_ the force at the end of the stretch, slope_1_ the initial rapid rise in force immediately after stretch initiation, and slope_2_ the slope calculated over the final 5% of the stretch.

### 2.4 Statistical analysis

A two‐way analysis of variance (ANOVA) was conducted to evaluate the effects of contraction velocity and treatment on the outcome variables (*s*_*i*_, *s*_*e*_, slope_1_ and slope_2_). Velocity had three levels (0.01, 0.1, 1 *v*_*0*_), and treatment had four levels (control, BDM, Bleb and BTS). The ANOVA tested (1) the main effect of velocity, (2) the main effect of treatment and (3) the velocity × treatment interaction. *Post hoc* pairwise comparisons with Bonferroni correction were performed if significant main effects or interactions were identified.

Data are presented as means ± SD unless otherwise stated. *F*‐ and *t*‐statistics with corresponding *P*‐values are reported, and significance was set at *P* < 0.05. Effect sizes for the ANOVA were classified as small (η^2^ < 0.06), medium (0.06 ≤ η^2^ ≤ 0.14) and large (η^2^ > 0.14), and for paired Student's *t*‐test as small (*d* < 0.5), medium (0.5 ≤ *d* ≤ 0.8) or large (*d* > 0.8) (Cohen, 1998).

Temporal differences in continuous force data were assessed using statistical parametric mapping (SPM; Serrien et al., 2019) to compare the four treatments (control, BDM, Bleb and BTS) in SOL and EDL at the three stretch velocities tested. SPM analyses were conducted using the MATLAB package spm1d (Pataky, 2012), enabling continuous comparisons across the entire force–length–time course and identification of early‐phase (*s*_*i*_, slope_1_) and late‐phase (*s*_*e*_, slope_2_) differences. Force–length–time traces were interpolated to a uniform number of points before analysis to ensure consistent sampling.

## 3 RESULTS

Two‐way ANOVA revealed muscle‐ and velocity‐specific effects of treatment on all force parameters tested (*s*_*i*_, slope_1_, slope_2_, *s*_*e*_). In the SOL, isometric force (*s*_*i*_) was strongly affected by treatment (*F*(3,141) = 12900.752, *P* < 0.001, η*^2^* = 0.996), whereas velocity (Appendix, Figure A1a) and the velocity × treatment interaction were not significant; *post hoc* results are shown in Figure 1a and Table 1. In the EDL, treatment also significantly affected *s*_i_ (*F*(3,146) = 2290.070, *P* < 0.001, η^2^ = 0.975), with a significant velocity × treatment interaction (*F*(6,146) = 5.097, *P* < 0.001, η^2^ = 0.004), while the main effect of velocity was not significant (Appendix Figure A2e); *post hoc* results are shown in Figure 1c and Table 3. For slope_1_, slope_2_ and *s*_*e*_, significant main effects of velocity and treatment were observed in both muscles (*P* < 0.001; Appendix Figure A2), with interactions for slope_1_ and *s*_*e*_ in both muscles (*P* < 0.001) and for slope_2_ in the SOL (*P* < 0.05) (*post hoc* results shown in Figures 1b, d and 2; Tables 1, 2, 3, 4).

Muscle‐specific differences were evident in the effects of the inhibitors on *s*_*i*_. In the SOL, XB inhibition was strongest with Bleb (0.05 ± 0.04*F*_*0*_; Figure 1a, yellow), followed by BDM (0.12 ± 0.02*F*_*0*_; Figure 1a, red), whereas BTS produced the highest isometric force (0.37 ± 0.03*F*_*0*_; Figure 1a, blue), indicating the weakest XB inhibition. In the EDL, XB inhibition was strongest with BTS (0.005 ± 0.02*F*_*0*_; Figure 1c, blue) and Bleb (0.01 ± 0.01*F*_*0*_; Figure 1c, yellow), while BDM showed intermediate inhibition and the highest *s*_*i*_ (0.15 ± 0.11*F*_*0*_; Figure 1c, red).

Analysis of continuous force–time data using SPM supported the discrete parameter results. All inhibitors reduced force across the entire stretch range relative to control (*P* < 0.001; Figure 3), with force generally increasing during stretch. In the SOL, particularly under BTS at higher velocities, pronounced muscle ‘*give*’ was evident (Figure 3a, right panel), consistent with detachment of remaining active XBs, which persisted at 37% of *F*_*0*_ under BTS. When XB‐associated ‘*give’* occurred, remnants of short‐range stiffness (slope_1_) and a clear separation between early and late stretch phases were observed. In contrast, the absence of ‘*give*’ was characterised by a more exponential increase in force (Figure 3b, e.g., yellow trajectory).

The eccentric force response is velocity‐dependent. At slow velocities (0.01*v*_*0*_), approximate scaling with *s_i_* was observed – particularly in the SOL – as force–time–length trajectories ran nearly parallel to each other and to control (cf. coloured vs. black curves in Figure 3), consistent with significant *s_i_* differences between BTS and the other inhibitors. At the highest velocity (1 *v*_*0*_), pronounced length‐dependent differences emerged, with force–time–length curves crossing. Notably, under Bleb, which produced the lowest *s_i_* in the SOL across all velocities and inhibitors (*P* < 0.001; Figures 1a and 3a, yellow), force became the largest by the end of the stretch at high velocity compared with BDM (*s*_*e*_; *P* < 0.001; Figures 1b and 3a, right panel), indicating inhibitor‐specific modulation of both XB and non–XB force contributions.

SPM{*F*} analyses confirmed significant differences among inhibitors across velocities in both muscles (Figure 4). In the SOL, all three inhibitors differed throughout the entire force–length–time course at 0.01, 0.1 and 1*v*_*0*_ (all *P* < 0.001; Figure 4a), with velocity‐dependent pairwise differences evident across early and late stretch phases (Figure 5a). In the EDL, inhibitor effects were more temporally restricted: differences occurred from 0 to 75% of stretch at 0.01*v*_*0*_ (*P* < 0.001), from 0 to 60% at 0.1*v*_*0*_ (*P* = 0.002), and were confined to the early phase (0–15%) at 1*v*_*0*_ (*P* = 0.029) (Figure 4b). Pairwise comparisons showed that BDM differed from both Bleb and BTS during early stretch, whereas Bleb and BTS did not differ significantly in the EDL at any velocity (Figure 5b). Stretch velocity and the type of XB inhibitor influenced *s*_*e*_ in both SOL and EDL fibres. However, in EDL fibres, *s*_*e*_ did not differ significantly between XB inhibitors (Figure 1d; Table 3) and exhibited velocity‐dependent effects under Bleb and BTS only, but not under BDM (Appendix Figure A2f). In contrast to slope_1_, slope_2_ was largely preserved in both muscles under XB inhibition and, in some conditions, matched or exceeded control values, particularly at higher velocities (Figure 2b,d; Appendix Figure A2d,h; Tables 2 and 4).

## 4 DISCUSSION

Our study systematically examined the effects of commonly used chemical myosin II XB inhibitors on the mechanics of striated skeletal muscle during prolonged active stretches. Consistent with previous findings, we show that BDM, Bleb and BTS substantially reduce both calcium‐activated isometric force and eccentric force in skinned single fibres during long isokinetic stretches of 45% *L*_*0*_. This reduction was observed across all tested stretch velocities (0.01, 0.1, 1*v*_*0*_), and in both fast‐twitch (EDL) and slow‐twitch (SOL) fibres. The two main findings of this study are: (1) chemical XB‐inhibitors differentially affect eccentric force (*s*_*e*_) and slope_2_ in fast versus slow‐twitch muscle; and (2) the effects on all four force parameters (*s*_*i*_, slope_1_, slope_2_, *s*_*e*_) vary according to the specific combination of inhibitor, fibre type and stretch velocity, indicating distinct interaction‐dependent mechanical responses.

While the sample size in some groups (e.g., BTS‐treated SOL, *n* = 4) is relatively small, this reflects the technical and time‐intensive nature of single‐fibre experiments. Despite this limitation, the observed effects were consistent and in agreement with existing literature (Bond et al., 2013). Given the controlled experimental design and the clear and distinguishable differences between conditions, the sample size appears to be sufficient to address the mechanistic aims of the study.

### 4.1 Onset‐of‐stretch force (*s*_*i*_)

In EDL fibres, all three inhibitors strongly suppressed isometric force (*s*_*i*_), though not completely, at concentrations of 10 mM BDM, 20 µM Bleb and 50 µM BTS, respectively, reducing *s*_*i*_ to ∼5–18% of control, with BDM producing slightly less effective XB inhibition. These results align with previous biochemical, crystallographic and biomechanical studies in skinned rabbit skeletal single fibres using BDM, BTS and Bleb (Iwamoto, 2018), rat EDL fibres using BDM (Fryer et al., 1988; Tomalka et al., 2017), and rabbit psoas myofibrils using Bleb (Shalabi et al., 2017).

In the SOL, inhibition was strongest with Bleb (∼5% *F*_*0*_), followed by BDM (∼12% *F*_*0*_), while BTS preserved the highest *s*_*i*_ (∼37% *F*_*0*_). These results align with previous studies on skinned rat SOL fibres (Fryer et al., 1988; Tomalka et al., 2020, 2021). The limited reduction observed with BTS (∼40% of control) is consistent with its selective inhibition of fast‐twitch myosin II (Bond et al., 2013; Cheung et al., 2002; Ramachandran et al., 2003). Collectively, these findings highlight both muscle‐type and inhibitor‐specific differences in XB‐mediated force suppression.

### 4.2 Effects of XB‐inhibitors during active‐lengthening

#### 4.2.1 Early‐stretch force development (slope_1_)

All three inhibitors markedly suppressed the characteristic XB‐mediated features in the short‐range of the stretch (up to ∼1.5% *L*_*0*_, defined by slope_1_; cf. Weidner et al., 2024), with the magnitude of suppression differing between muscles (Figures 2 and 3). In inhibitor‐treated fibres, slope_1_ was largely insensitive to stretch velocity in both SOL and EDL, except for BTS in the SOL (*P* < 0.05; Appendix Figure A2). Under control conditions, slope_1_ increased systematically with velocity in both muscles (*P* < 0.001), consistent with previous reports (Burmeister Getz et al., 1998; Pinniger et al., 2006; Tomalka et al., 2021; Weidner et al., 2022, 2024). This velocity dependence is classically attributed to short‐range stiffness, which reflects the slightly damped elastic resistance of active muscle to small, rapid length perturbations over the first ∼1–2% of fibre length change (Campbell & Lakie, 1998; Ford et al., 1977; Morgan, 1977; Rack & Westbury, 1974). This response is thought to include a viscous component (Cecchi et al., 1997), potentially arising from the myosin S2 region or from non‐XB structural elements (Mutungi & Ranatunga, 1996). Chemical XB inhibition largely abolished this velocity‐dependent behaviour, indicating that short‐range stiffness is predominantly XB‐mediated. The residual velocity dependence observed with BTS in the SOL (*P* < 0.05; Appendix Figure A2c) likely reflects incomplete inhibition of slow myosin isoforms.

Quantitatively, inhibitors reduced slope_1_ in SOL fibres by approximately one order of magnitude compared to the control (Figure 2a; Table 2). In EDL fibres, reductions were more pronounced, reaching nearly two orders of magnitude with Bleb and BTS and approximately one order of magnitude with BDM (Figure 2c; Table 4). The suppression – and in some cases near elimination – of the XB‐mediated initial steep force rise was accompanied by a marked reduction in the negative force slope associated with muscle ‘*give*’ following slope_1_, consistent with previous findings in SOL and EDL fibres (Pinniger et al., 2006; Tomalka et al., 2017, 2020, 2021).

#### 4.2.2 Late‐stretch force development (slope_2_)

Despite strong suppression of slope_1_, fibres maintained substantial force development during the later stretch phase. Under control conditions, slope_2_ increased quasi‐linearly with stretch velocity in both EDL and SOL, consistent with previous reports (Figure 3; Till et al., 2008; Tomalka et al., 2017, 2021; Weidner et al., 2022, 2024). slope_2_ was largely preserved under XB inhibition (Figure 2b,d; Appendix Figure A2b,d; Tables 2 and 4), indicating that late‐phase force development is modulated by muscle type, treatment and velocity, and is not primarily determined by strongly bound XBs. Rather, late‐phase force development is likely dominated by non‐XB‐mediated mechanisms, including titin‐based stiffness (Colombini et al., 2016; Hessel, Kuehn et al., 2024; Nocella et al., 2014), contributions from weakly bound XBs (Colombini, Nocella, Bagni et al., 2010; Iwamoto, 2018; Pinniger et al., 2005), and myofilament lattice interactions (Hessel et al., 2022, 2024b).

When XB‐mediated short‐range stiffness is suppressed (slope_1_), non‐XB structures bear an increased fraction of the imposed stretch, shaping both the magnitude and trajectory of force development during the later stretch phase. Accordingly, velocity‐dependent increases in slope_2_ were largely maintained across treatments and muscles (Appendix Figure A2d,h), except for BDM‐treated EDL fibres, where this relationship was attenuated (Appendix Figure A2h, red). Because XB‐generated forces typically decline on the descending limb of the force–length relationship, the observed positive slope_2_ and its velocity‐dependent increase (Figure 2b,d; Tables 2 and 4) are consistent with a strong, linear viscoelastic contribution from titin (Chung et al., 2011; Hessel, Kuehn et al., 2024). Collectively, these findings support the interpretation that slope_2_ reflects a largely XB‐independent, late‐phase component of eccentric force that is unmasked when short‐range XB stiffness is suppressed.

#### 4.2.3 End‐of‐stretch force (*s*_*e*_)

Eccentric forces at the end of the stretch were reduced proportionately less than *s*_*i*_, consistent with previous studies (Minozzo & Rassier, 2010; Rahman et al., 2018; Rassier & Herzog, 2004; Roots et al., 2007; Tomalka et al., 2017). When normalized to individual *s*_*i*_, forces at the end of the stretch (*s*_*e*_) were markedly amplified relative to controls (factor 1.3–2.4 in controls, up to ∼290 in inhibited fibres), particularly at higher velocities and in fast‐twitch fibres (Figure 3). This amplification indicates a substantial contribution from non‐XB elements to force transmission during eccentric contractions, with titin as the main contributor (Hessel, Kuehn et al., 2024; Tomalka, 2023).

These relationships demonstrate that XB inhibition alters not only early force generation but also the magnitude and velocity dependence of sustained stretch. Velocity‐dependent variability in *s*_e_ was greater in the EDL than in the SOL, as reflected by the larger standard deviations observed in fast‐twitch fibres (Figure 3; Appendix Figure A2b,f; Tables 1 and 3). Such elevated variability in fast‐twitch fibres has been reported previously in both single fibres and fibre bundles (Prado et al., 2005; Weidner et al., 2022, 2024) and likely reflects heterogeneity in passive structural elements and titin isoforms in fast‐twitch fibres. This explanation is further supported by evidence that non‐XB contributions to eccentric force increase with muscle length (Rode et al., 2009; Tomalka et al., 2017) and are particularly pronounced under XB inhibition (Colombini et al., 2016; Tomalka et al., 2017). In addition, the expression of two myosin heavy‐chain isoforms in the EDL (Ausoni et al., 1990; Soukup et al., 2002) may further contribute to the observed variability in *s*_*e*_.

### 4.3 Effects of chemical agents on XB and non‐XB structures

The present study directly compares the effects of BDM, Bleb and BTS with one another and with control trials across fibre types and stretch velocities. This approach reveals distinct quantitative differences in force trajectories and demonstrates that each inhibitor uniquely modulates fibre mechanics through differential effects on XB and non‐XB structures.

### 4.4 Influence of BDM

Previous findings by Tomalka et al. (2017) on skinned rat EDL fibres during prolonged stretches at 0.1*v*_*0*_ demonstrated that deviations between scaled isometric XB forces during ramp contractions and the theoretical sarcomere force–length relationship increased with higher BDM concentrations (see Figure 3b in Tomalka et al., 2017), suggesting that BDM may also affect non‐XB force components. Comparable observations have been reported in intact frog muscle fibres subjected to stretches of 5% and 10% of fibre length in the presence of 2, 5 and 10 mM BDM (Rassier & Herzog, 2004). However, these observations remained tentative due to the lack of systematic approaches separating XB and non‐XB contributions during prolonged stretches.

Mechanistically, BDM stabilises myosin heads in a weakly bound, pre‐power stroke state, thereby reducing the number of strongly attached XBs during stretch. Acting directly and reversibly on the myosin heads at the myofibrillar level (Higuchi & Takemori, 1989), BDM slows myosin ATPase activity of skeletal muscle myosin II by hindering inorganic phosphate (Pi) release even at low BDM concentrations (<1 mM) (Bagni et al., 1992; Bond et al., 2013; Higuchi & Takemori, 1989; McKillop et al., 1994). Consistent with these mechanisms, BDM moderately reduced isometric force (*s*_*i*_: SOL ∼12% *F*_*0*_; EDL ∼15% *F*_*0*_) and suppressed the early‐phase force rise (slope_1_). In contrast, late‐phase force development (slope_2_) was largely preserved across velocities in SOL and EDL (Figure 2b,d; Appendix Figure A2d,h), reflecting prominent non‐XB contributions.

Despite its utility, BDM exhibits limited specificity and broad off‐target effects (Bond et al., 2013; Ramachandran et al., 2003). In addition to inhibiting skeletal muscle myosin II, BDM can interfere with myosin light chain kinase activity (Siegman et al., 1994), neurotransmitter release (Sellin & McArdle, 1994), voltage‐ and ligand‐activated ion channels in muscle and nerve cells (Sellin & McArdle, 1994), protein phosphorylation in cardiomyocytes (Stapleton et al., 1998) and calcium regulation (Fryer et al., 1988). Notably, BDM also affects protein phosphorylation (Sellin & McArdle, 1994; Stapleton et al., 1998) and calcium‐dependent regulation (Fryer et al., 1988), which modulate XB kinetics and the availability of actin‐binding sites on the thin filament, effectively acting as an on–off switch for contraction (Schiaffino, 2018; Schiaffino & Reggiani, 2011). These off‐target effects, combined with its low binding affinity, must be carefully considered when interpreting results obtained with BDM, particularly in vivo.

### 4.5 Influence of BTS

In contrast to BDM, BTS selectively inhibits fast myosin II with >100‐fold higher potency than slow skeletal, cardiac or non‐muscle myosin II (Cheung et al., 2002), slowing Pi and ADP release and hindering actin binding (Bond et al., 2013; Cheung et al., 2002; Shaw et al., 2003). In our data, BTS nearly abolished *s*_*i*_ in fast‐twitch EDL (∼0.5% *F*_*0*_) but produced substantially less inhibition in SOL (∼37% *F*_*0*_; Figure 1a,c), highlighting fibre‐type specificity. This selectivity is clearly reflected in the present data, where BTS produced markedly stronger inhibitory effects in fast‐twitch EDL fibres than in slow‐twitch SOL fibres (Figure 3, blue trajectories). This pronounced fibre‐type dependence reflects the limited sensitivity of slow myosin isoforms to BTS and indicates that, in slow‐twitch muscle, BTS is not an appropriate tool for selectively probing XB kinetics or isolating non‐XB contributions during stretch. Importantly, BTS is reversible, does not alter Ca^2^^+^ dynamics (Cheung et al., 2002; Pinniger et al., 2005; Ramachandran et al., 2003), and does not affect myosin or actin expression levels (Ramachandran et al., 2003).

### 4.6 Influence of Bleb

Bleb, one of the most widely used and extensively characterized small‐molecule myosin II inhibitors, suppresses force production by blocking a critical transition in the myosin ATPase cycle and thereby exerts complex effects on XB function (Bond et al., 2013). Specifically, Bleb selectively inhibits the transition from weakly to strongly bound states (Iwamoto, 2018; Rahman et al., 2018), effectively trapping myosin heads in a weakly bound ADP·Pi state that does not generate force (Kovács et al., 2004).

Consistent with this mechanism, Bleb produced near‐complete suppression of early‐phase XB‐mediated force (*s*_*i*_ and slope_1_) while preserving the structural presence of attached myosin heads, making it particularly useful for dissecting the contributions of force‐generating versus non‐force‐generating XB states during stretch. Across all velocities and treatments tested, Bleb (and BTS in EDL) induced the largest reductions in isometric force (*s*_*i*_; SOL: ∼5% *F*_*0*_; EDL: ∼1.5% *F*_*0*_) and strongly suppressed slope_1_, yet late‐phase force development (slope_2_) was largely preserved and exceeded control values in both SOL and EDL at every velocity, indicating prominent contributions from non‐XB structures (Figure 2b,d; Appendix Figure A2d,h; Tables 2 and 4). Maximal eccentric forces at the end of stretch (*s*_*e*_) were markedly amplified relative to individual *s*_*i*_ (SOL: up to ∼137% *F*_*0*_; EDL: ∼195% *F*_*0*_; Figure 3), particularly at higher velocities and in fast‐twitch fibres, consistent with titin‐mediated force transmission.

Although Bleb's inhibitory effects are reversible, it is highly photosensitive and undergoes rapid degradation upon exposure to light in the 365–490 nm range (Sakamoto et al., 2005), which must be carefully controlled during experiments.

### 4.7 Challenges in mechanistic interpretation

The agent‐specific molecular actions of the XB inhibitors likely underlie the qualitative and quantitative differences observed in force trajectories during active muscle lengthening, reflecting how each compound differentially modulates XB kinetics and the engagement of non–XB structural elements. Providing a unified mechanistic explanation for the eccentric force responses remains challenging, as inhibitor effects arise from multiple interacting factors, including altered XB attachment–detachment kinetics, non‐linear effects on force generation (e.g., non‐XB force‐length relationship), and alterations in effective myofilament overlap (Bond et al., 2013; Rahman et al., 2018; Rassier & Månsson, 2025; Tomalka et al., 2017).

Muscle‐specific differences in force profiles further reflect variations in fibre‐type composition and titin isoforms. Slow‐twitch (SOL) and fast‐twitch (EDL) muscles differ structurally and functionally, with pronounced differences in XB dynamics largely determined by myosin heavy‐chain (MHC) isoform expression (Capitanio et al., 2006; Pellegrino et al., 2003). In rats, MHC isoforms define the principal fibre types (I, IIa, IIb, IIx), while three regulatory myosin light‐chain (MLC) isoforms – two fast (MLC‐1f, MLC‐3f) and one slow (MLC‐1s) – are differentially distributed across fibres (Jones et al., 2004; Schiaffino & Reggiani, 1994). Because MHC and MLC composition governs myosin ATPase activity, XB turnover and energetic cost (Pellegrino et al., 2003; Schiaffino & Reggiani, 2011), pharmacological inhibition of myosin II ATPase is expected to produce fibre‐type‐specific effects on both isometric and eccentric force generation.

Skeletal muscles also express distinct titin isoforms, with substantial variation in the length of the N2A and PEVK regions (Labeit & Kolmerer, 1995; Prado et al., 2005), which strongly influence muscle‐specific mechanical properties (Freiburg et al., 2000; Linke, 2018; Prado et al., 2005). Fast muscles, characterised by high proportions of fast MHCs, predominantly express shorter, stiffer N2A titin variants, whereas slow skeletal muscles express longer N2A isoforms, as shown in rabbit muscle (Prado et al., 2005). These structural differences likely contribute to the muscle‐type‐specific force responses observed during active stretch. Shorter titin isoforms in fast muscles may enhance titin‐mediated force during and after eccentric contractions, particularly when XB‐mediated force is suppressed (Rassier & Månsson, 2025). This aligns with the larger relative eccentric forces and greater variability observed in fast‐twitch fibres in the present study. Additionally, titin stiffness is extensively regulated by phosphorylation at over 300 sites via multiple kinases (Loescher et al., 2022), and BDM's inhibition of myosin light‐chain kinase may further modulate fibre mechanics and force development during stretch (Bond et al., 2013; Siegman et al., 1994).

### 4.8 Conclusions

Although Bleb, BDM and BTS are commonly classified as specific inhibitors of skeletal muscle myosin II (Bond et al., 2013), their effects likely extend beyond simple ATPase inhibition or the prevention of actomyosin interaction. Bleb was the most potent XB inhibitor in both slow‐twitch SOL and fast‐twitch EDL fibres. Although it markedly suppressed isometric force (*s*_*i*_), Bleb produced the highest eccentric force (*s*_*e*_). One interpretation of these results is that non‐XB contributions were preserved or even enhanced during active stretch.

Overall, these findings highlight Bleb as the most effective tool for probing the relative contributions of XB and non‐XB structures during prolonged active stretches. However, several experimental constraints, including temperature sensitivity, fibre preparation and potential off‐target effects, underscore the need for further studies to understand the mechanistic basis of these differential inhibitory effects fully. Future studies should also examine clinically relevant, more selective myosin inhibitors, such as mavacamten, to better characterise their impact on muscle performance.

## AUTHOR CONTRIBUTIONS

Conceptualisation, André Tomalka, Sven Weidner, Christian Rode, Tobias Siebert; methodology, André Tomalka; formal analysis, André Tomalka, Sven Weidner; investigation, André Tomalka; resources, André Tomalka, Tobias Siebert; writing – original draft preparation, André Tomalka; writing – review and editing, André Tomalka, Sven Weidner, Tobias Siebert; visualisation, André Tomalka; funding acquisition, André Tomalka, Christian Rode, Tobias Siebert All authors approved the final version of the manuscript. They agree to be accountable for all aspects of the work in ensuring that questions related to the accuracy or integrity of any part of the work are appropriately investigated and resolved; and all persons designated as authors qualify for authorship, and all those who qualify for authorship are listed.

## CONFLICT OF INTEREST

None declared.

5

## Data Availability

The data presented in this study are available on request from the corresponding author.

## Fibre handling, preparation and measurement

The methods used for muscle preparation, storage and activation of permeabilised single muscle fibres followed the protocols described by Tomalka et al. (2017, 2024). Briefly, glycerinated skinned single fibre segments were obtained from the EDL muscles of five female Wistar rats (age: 8–10 months, weight: 300–350 g) and the SOL of six freshly euthanised female Wistar rats (age: 3 months, weight: 428–520 g). The rats were cage‐sedentary, maintained on a 12 h–12 h light–dark cycle and housed at a temperature of 22°C. The use of skeletal muscle fibres from rats was approved in accordance with the German animal protection law (Tierschutzgesetz, §4 (3); Permit Number: T 201_21 ST). After permeabilisation in a skinning solution, the muscle fibre bundles were stored at −20°C in a storage solution (cf. Table 1 in Tomalka et al. (2017)).

On the day of the experiments, small segments of the skinned fibre bundles were carefully dissected, and single muscle fibres were isolated. The fibre bundles were then treated with a relaxing solution (see Table 1 in Tomalka et al. (2017)) containing Triton X‐100 (1% v/v) for 2–3 min at 4°C. This treatment effectively removed internal membranes without disrupting the contractile apparatus (Linari et al., 2007). Each end of the single muscle fibre was attached to a hook connected to a force transducer (model 403a, Aurora Scientific, Aurora, ON, Canada) and a high‐speed length controller (model 322C‐I, Aurora Scientific, Aurora, ON, Canada), respectively. During the kinetic experiments, sarcomere length (SL) was monitored using a high‐speed camera system (model 901B, Aurora Scientific, Aurora, ON, Canada) coupled with a ×20 ELWD dry objective (NA 0.40, Nikon, Tokyo, Japan) and an accessory lens (×2.5, Nikon, Tokyo, Japan). The average length of the passive sarcomeres within each fibre was set to 2.5 ± 0.05 µm, defined as the optimal fibre length (*L*_*0*_) for each experiment. Fibre width (*w*) and height (*h*) were measured at three positions along the fibre segment in the relaxed state using a ×10 dry objective (NA 0.30, Nikon, Tokyo, Japan) and a ×10 eyepiece. The three measurements were averaged for each dimension. The fibre cross‐sectional area (CSA) was calculated assuming an elliptical cross‐section for the single muscle fibres (Mebrahtu et al., 2024). The CSA was 0.0045 ± 0.0018 mm^2^ for the EDL and 0.006 ± 0.002 mm^2^ for the SOL (mean ± standard deviation). All experiments were conducted at a temperature of 12 ± 0.1°C.

## Solutions

The relaxing solution contained (in mmol l^−1^): 100 TES, 7.7 MgCl_2_, 5.44 Na_2_ATP, 25 EGTA, 19.11 Na_2_CP and 10 glutathione (pCa 9.0). The pre‐activating solution contained (in mmol l^−1^): 100 TES, 6.93 MgCl_2_, 5.45 Na_2_ATP, 0.1 EGTA, 19.49 Na_2_CP, 10 glutathione and 24.9 HDTA. The activating solution contained (in mmol l^−1^): 100 TES, 6.76 MgCl_2_, 5.46 Na_2_ATP, 19.49 Na_2_CP, 10 glutathione and 25 CaEGTA (pCa 4.5). The skinning solution contained (in mmol l^−1^): 170 potassium propionate, 2.5 MgCl_2_, 2.5 Na_2_ATP, 5 EGTA, 10 imidazole and 0.2 phenylmethylsulfonyl fluoride. The storage solution was the same as the skinning solution, except for the presence of 10 mmol l^−1^ glutathione and 50% glycerol (*v/v*). Cysteine and cysteine/serine protease inhibitors (*trans*‐epoxysuccinil‐l‐leucylamido‐(4‐guanidino) butane, E‐64, 10 mmol l^−1^; leupeptin, 20 µg ml^−1^) were added to all solutions to preserve lattice proteins and thus sarcomere homogeneity (Linari et al., 2007; Tomalka et al., 2017). pH (adjusted with KOH) was 7.1 at 12°C. Creatine phosphokinase (450 U/ml^−1^) was added to all solutions, except for skinning and storage solutions. Creatine phosphokinase was obtained from Roche (Mannheim, Germany). Bleb was obtained from Enzo Life Sciences Inc. (NY, USA), BDM, BTS and all other chemicals were obtained from Sigma (St Louis, MO, USA).

Figure A1.

Figure A2.
